# Therapeutic exercise is associated with dose-dependent reductions in acute anxiety across psychiatric diagnoses

**DOI:** 10.3389/fpsyt.2026.1805126

**Published:** 2026-03-18

**Authors:** Sandra Perunova, Inese Kokina, Indrikis A. Krams

**Affiliations:** 1 Daugavpils Psychoneurological Hospital, Daugavpils, Latvia; 2 Department of Biodiversity, Institute of Life Sciences and Technology, Daugavpils, Latvia; 3 Department of Technology, Institute of Life Sciences and Technology, Daugavpils, Latvia; 4 Latvian Biomedical Research and Study Centre, Riga, Latvia; 5 Department of Ecology, Faculty of Medicine and Life Sciences, University of Latvia, Riga, Latvia

**Keywords:** aerobic walking, anxiety reduction, diaphragmatic breathing, GAD-7 assessment, psychiatric patients, stretching and relaxation, therapeutic exercise

## Abstract

**Introduction:**

Acute anxiety is common across psychiatric disorders and may interfere with treatment engagement. Although physical activity is known to improve long-term mental health outcomes, less is known about its immediate associations with anxiety reduction in real-world psychiatric settings. This study examined whether participation in therapeutic exercise sessions is associated with short-term changes in self-reported anxiety and whether greater program participation is related to larger reductions.

**Materials and methods:**

We analyzed data from 179 adult psychiatric patients participating in a structured therapeutic exercise program including (i) 30–45-minute walking sessions, (ii) stretching/yoga-like sessions, and (iii) 5–10-minute diaphragmatic breathing exercises. Self-reported anxiety was assessed using the GAD-7 questionnaire administered immediately before and immediately after exercise sessions. Non-parametric tests evaluated pre–post changes, and Spearman correlations assessed associations between session counts and anxiety change.

**Results:**

Self-reported anxiety scores were significantly lower after exercise sessions (median pre = 3; median post = 2; Wilcoxon P < 0.001). Improvement was observed in 81% of patients. Anxiety reductions did not differ significantly by sex, diagnosis, or age. Greater participation in walking, stretching/yoga, and breathing sessions was associated with larger decreases in anxiety (Spearman ρ = −0.63 to −0.66, all P < 0.001). Because exercise modalities were highly intercorrelated, these findings likely reflect overall program engagement rather than modality-specific effects.

**Discussion:**

In this observational clinical sample, participation in exercise sessions was consistently associated with large, rapid short-term reductions in self-reported anxiety across diagnostic categories. The observed dose–response associations suggest that greater engagement may be linked to larger improvements. However, the absence of a control group and the use of a symptom questionnaire in a session-based format limit causal inference. Controlled trials are needed to determine the extent to which exercise itself, expectancy effects, or contextual factors contribute to these improvements.

## 1 Introduction

Anxiety symptoms are highly prevalent across psychiatric diagnoses—including schizophrenia, bipolar disorder, major depressive disorder, and generalized anxiety disorder—and contribute substantially to functional impairment, hospitalization, and treatment non-adherence (1–3). Despite therapeutic advances, clinicians often face challenges managing episodes of acute anxiety that emerge during treatment or rehabilitation. This has led to increasing interest in non-pharmacological interventions that act rapidly, safely, and can be implemented repeatedly across diagnostic categories.

Physical activity is widely recognized as beneficial for mental health, with robust evidence showing that chronic exercise programs (lasting weeks to months) reduce anxiety, depression, and physiological stress reactivity (4, 5). However, fewer studies address acute, session-by-session anxiolytic effects. Meta-analytic evidence indicates that a single bout of exercise can reduce state anxiety (6–12), but most studies are limited by small samples and focus on single diagnostic categories, especially generalized anxiety or depression. Consequently, it remains unclear whether therapeutic exercise is associated with rapid reductions in anxiety across a heterogeneous psychiatric population.

Transdiagnostic improvement is theoretically plausible. Contemporary frameworks such as the National Institute of Mental Health’s Research Domain Criteria (RDoC) emphasize that abnormalities in arousal regulation, autonomic control, and somatic tension contribute to psychopathology across diagnostic boundaries (13, 14). These processes are strongly influenced by physical activity, stretching, and controlled breathing—suggesting that exercise-based interventions may yield broad anxiolytic benefits regardless of diagnosis. Recent empirical findings are consistent with this view: for example, an online yin yoga intervention significantly reduced state anxiety in women, demonstrating rapid anxiolysis even in remote or low-intensity exercise formats (15).

The present study leverages a unique real-world clinical dataset from 179 psychiatric patients who regularly participated in a structured exercise program including (a) 45-minute walking sessions, (b) 45-minute stretching/yoga-like sessions, and (c) 5-minute diaphragmatic breathing exercises. Self-reported anxiety was recorded immediately before and after exercise, along with demographic and diagnostic information. This design enables us to test two central questions: (i) Do exercise sessions reliably reduce short-term self-reported anxiety across psychiatric diagnoses? (ii) Does the amount (“dose”) of exercise performed—walking, stretching, breathing—modulate the magnitude of improvement?

Our findings show a strong and consistent reduction in short-term self-reported anxiety immediately following exercise, independent of age, sex, or diagnosis. Importantly, we also identify clear dose–response associations: patients who completed more walking, stretching, or breathing sessions showed larger reductions in anxiety and lower post-exercise anxiety levels. These results support the view that exercise may serve as a rapid, transdiagnostic adjunctive strategy associated with anxiolytic effects, with potential for integration into routine psychiatric care.

## 2 Materials and methods

### 2.1 Participants and setting

Data were collected from 179 adult psychiatric patients who participated in a multidisciplinary therapeutic program at the Daugavpils Psychoneurological Hospital, Latvia, from May to November 2025. The sample characteristics and diagnostic categories are presented in **Tables 1, A, B**. Patients participated in structured, movement-based sessions as part of their routine clinical care. All assessments and exercises were conducted by trained clinical specialists. The study participants were 56.4% women and 43.6% men, with a mean age of 49.0 ± 14.7 years. All participants were inpatients receiving structured psychiatric treatment at the Daugavpils Psychoneurological Hospital during the study period. The exercise sessions were integrated into routine inpatient care.

**Table 1A:** Sample characteristics and self-reported anxiety scores (*n* = 179).

Variable	Value
Patients (*n*)	179
Age, mean (SD), years	49.0 (14.7)
Women, *n* (%)	101 (56.4%)
Men, *n* (%)	78 (43.6%)
Pre-exercise anxiety, median [IQR]	3 [3–3]
Post-exercise anxiety, median [IQR]	2 [1–2]
Improved/same/worse (Δ)	145/34/0

**Table 1B:** Diagnoses in the sample.

Diagnosis	*n*	%
Generalized anxiety disorder (GAD)	55	30.7
Major depression	44	24.6
Schizophrenia	36	20.1
Bipolar disorder (manic)	24	13.4
Bipolar disorder (depressive)	20	11.2

The study participants had established ICD-10 diagnoses of schizophrenia, bipolar affective disorders, depressive episode, or generalized anxiety, who had been prescribed psychotropic medications for at least one month, and who were engaged in physical activities. Somatic comorbidities were evaluated through routine clinical procedures, including review of medical records and physician-conducted health examinations prior to participation in the exercise program. Patients with unstable or clinically significant somatic conditions contraindicating moderate physical activity were not enrolled. No such contraindicating somatic conditions were identified among included participants. All participants were medically cleared for participation in moderate physical activity as part of routine clinical care.

All participants were receiving routine psychopharmacological treatment prescribed by their treating psychiatrists. Inclusion required stable medication for at least one month prior to study participation. Medication regimens were determined by treating psychiatrists as part of routine care and were not controlled by the study. Therefore, no medication and no medication changes were introduced as part of the exercise intervention protocol.

### 2.2 Study protocol

The exercise program consisted of three modalities—aerobic walking, stretching/yoga-like exercises, and diaphragmic breathing—adapted for group-based psychiatric treatment. Exercise intensity was maintained at light to moderate levels to ensure safety and accessibility for psychiatric inpatients with heterogeneous physical capacity. Intensity was monitored clinically using patient tolerance and the Borg Rating of Perceived Exertion scale. No objective physiological measures (e.g., heart rate monitoring) were employed.

For each participant, the total number of completed sessions in each modality was recorded. Sessions were held daily, primarily in the morning between 9:00 a.m. and 1:00 p.m. During the final sessions, patients were educated on how to continue physical activity independently.

Before the sessions, an examination of the patient’s general health status and functional condition is carried out. The patient’s initial assessment includes anamnesis, objective examination, and evaluation of cardiovascular exercise tolerance using functional and exercise scales (the Borg Scale and the 6-Minute Walk Test for assessing functional endurance).

### 2.3 Exercise modalities and duration

Aerobic exercises consisted of walking or Nordic walking performed daily with the duration of 30–45 min per session. Stretching and relaxation exercises included back, shoulder, and neck exercises with the frequency of 3–5 sessions per week, with the duration of 10–15 minutes per session. The equipment required: mat, stick, chair. The diaphragmatic breathing was performed at least twice daily, and the duration of 5–10 minutes per each session.

### 2.4 Details of therapeutic exercise program

The exercise program consisted of three modalities adapted for group-based psychiatric treatment. Sessions included up to five participants. For each participant, the total number of completed sessions in each modality—walking, stretching/yoga-like exercises, and diaphragmatic breathing—was recorded. These counts served as indices of exercise dose.

#### 2.4.1 Aerobic exercise (walking/Nordic walking)

Aerobic sessions were conducted daily and lasted 30–45 minutes. At the beginning of each session, clinicians instructed patients in proper breathing technique (inhale through the nose, exhale through the mouth). Participants walked at a self-selected pace while maintaining awareness of breathing, bodily sensations, and the surrounding environment.

According to clinical guidelines (source: Exercises and anxiety estimation), consistent moderate aerobic activity promotes endorphin release, reduces cortisol, and alleviates both acute and chronic anxiety.

#### 2.4.2 Stretching and relaxation exercises

In addition to the aerobic, stretching, and breathing components described above, patients participated in a structured exercise program designed specifically for individuals experiencing anxiety-related symptoms. All sessions used simple equipment (mat, stick, chair) and included three exercise categories: back exercises, shoulder and neck exercises, and standing stretching exercises.

Stretching, breathing exercises, and progressive muscle relaxation were delivered in 10–15-minute sessions targeting tension-prone areas (neck, shoulders, lower back). Movements included shoulder rolls, overhead arm extensions, and forward bends. Simple yoga-derived exercises—such as the cat–cow sequence—were used to mobilize the spine and regulate breathing. Participants synchronized movement with respiration (inhale into “cow,” exhale into “cat”) for 3–5 minutes. Additional standing stretches (squats, lunges, forward folds) were incorporated as tolerated.

Back exercises included three exercise types: supine full-body stretch, quadruped spinal mobility or cat-cow sequence, and knee-to-chest stretch (1). Supine full-body stretch: Patients lie on their backs with legs extended and toes pointing upward. During inhalation, they lengthened the body and stretched for approximately 5 seconds, then relaxed during exhalation. Performed 5 repetitions (2). Quadruped spinal mobility (cat–cow sequence): From a hands-and-knees position at shoulder width, patients inhaled while arching the spine downward and held the position for 5 seconds. They returned to neutral, exhaled, and rounded the spine upward for another 5 seconds. Performed 5 repetitions (3). Knee-to-chest stretch: In a supine position with legs extended, patients inhaled to lengthen the spine and exhaled while drawing one knee toward the chest with both hands, holding for 5 seconds, then extended the leg again. Repeated with the opposite leg. Performed 5 repetitions per leg.

Shoulder and neck exercises included seven exercise types: diaphragmatic breathing, shoulder elevation, neck rotation, shoulder rotations, arm elevation stretch, elbow circles, and chest expansion sequence (1). Diaphragmatic breathing (seated): seated with feet on the floor, patients placed one hand on the abdomen and the other on the sternum. They inhaled through the nose to expand the abdomen and exhaled through the mouth while keeping the chest still. Performed 10 repetitions (2). Shoulder elevation (seated): holding the sides of the chair, patients raised both shoulders, held them for 5 seconds, then lowered and relaxed. Performed 10 repetitions (3). Neck rotation: patients slowly turned their heads to the right, held for 5 seconds, then returned to the center and relaxed. The same movement was repeated to the left. Performed 10 repetitions per side (4). Shoulder rotations: with fingertips resting on the shoulders, patients performed circular movements forward 10 times and backward 10 times (5). Arm elevation stretch: holding opposite elbows, patients raised their arms during inhalation, held them for 5 seconds, and lowered them during exhalation. Performed 5 repetitions (6). Elbow circles: holding opposite elbows, patients performed circular arm movements 10 times in each direction (7). Chest expansion sequence: with arms raised to shoulder height and elbows bent, patients brought the arms together in front during exhalation, then opened them outward during inhalation, and held for 3 seconds before returning to the starting position.

Standing stretching exercises included 5 exercise types: overhead stick press, rowing motion with a stick, behind-the-back arm extension, squats, and forward lunges (1). Overhead stick press: holding a stick overhead, patients bent their elbows to bring the stick to their shoulders, straightened their elbows to raise it forward, and then lowered it. Performed 10 repetitions (2). Rowing motion with a stick: holding the stick with both hands, patients mimicked rowing movements forward and backward. Performed 10 repetitions in each direction (3). Behind-the-back arm extension: with arms extended behind the back holding the stick, patients bent and straightened the elbows while keeping the torso upright. Performed 10 repetitions (4). Squats: standing with feet hip-width apart, patients inhaled deeply, engaged the abdominal muscles, and initiated the squat by bending the knees, hips, and ankles simultaneously. Heels remained on the floor. They rose slowly while exhaling. Performed 5–8 repetitions (5). Forward lunges: from an upright stance, patients stepped forward with one leg and lowered their body until both knees reached approximately 90°. They pushed through the front leg to return to standing, inhaling during descent and exhaling on return. Repeated with the opposite leg. Performed 5–10 repetitions.

#### 2.4.3 Diaphragmatic breathing

Controlled breathing exercises were used to activate the parasympathetic nervous system and reduce autonomic arousal. Diaphragmatic breathing consisted of 5–10-minute sessions performed at least twice daily.

Participants were guided to slow their exhalation to enhance vagal activity and promote emotional regulation. Breathing exercises were included in every exercise session (4, 7, 16).

### 2.5 Anxiety assessment

The GAD-7 was originally developed to assess generalized anxiety symptoms over the preceding two weeks. In the present study, the instrument was administered immediately before and after exercise sessions in a repeated, session-based format to capture short-term changes in self-reported anxiety. This usage does not constitute formal validation as a state anxiety instrument and should be interpreted accordingly.

Self-reported anxiety was assessed using the Latvian version of the Generalized Anxiety Disorder 7-item scale (GAD-7). The Latvian adaptation (17) is a linguistically and psychometrically validated translation of the original instrument developed by Spitzer et al. (18), preserving its structure, scoring, and diagnostic thresholds.

The GAD-7 consists of seven items rated on a 0–3 scale (“not at all” to “nearly every day”), yielding total scores from 0 to 21. Standard cut-offs categorize anxiety as mild (5–9), moderate (10–14), or severe (15–21).

Patients completed the scale twice per session—immediately before and immediately after the exercise intervention—to assess short-term changes in self-reported anxiety. At baseline, patients typically endorsed elevated symptoms such as nervousness, tension, excessive worry, and catastrophic thoughts; these symptoms showed measurable reductions following exercise sessions.

### 2.6 Statistical analysis

Given the ordinal nature and non-normal distribution of the data, non-parametric methods were used. Analyses included: Wilcoxon signed-rank tests for pre–post self-reported anxiety comparisons; Sign tests to evaluate direction-only improvement; Kruskal–Wallis and Mann–Whitney tests for diagnostic and demographic moderators; Spearman correlations to assess dose–response associations.

In addition to correlation analyses, we performed linear regression models as sensitivity analyses to quantify dose–response associations using total session count (walking + stretching/yoga + breathing) as the primary exposure. Models were estimated both unadjusted and adjusted for age, sex, and diagnosis. Regression coefficients are reported with 95% confidence intervals. Because residual variance and homoscedasticity could not be assumed, we used heteroskedasticity-robust standard errors (HC3). For regression models, we report R² and standardized coefficients; partial R² was used to summarize the incremental variance explained by total session count in adjusted models.

## 3 Results

### 3.1 Sample characteristics

The final dataset included 179 psychiatric patients (56.4% women; mean age 49.0 ± 14.7 years). Diagnoses were generalized anxiety disorder (GAD; 30.7%), major depression (24.6%), schizophrenia (20.1%), bipolar disorder – manic (13.4%), and bipolar disorder – depressive (11.2%) (**Table 1B**).

Pre-exercise self-reported anxiety scores were high and tightly clustered (median = 3 [IQR 3–3]), corresponding to severe anxiety on the clinical scale used. This concentration at the upper category indicates restricted baseline variance and potential ceiling compression inherent to the categorical severity scale. Such compression limits upward variability and may reduce sensitivity for detecting potential worsening. Post-exercise scores were lower and more variable (median = 2 [IQR 1–2]), indicating a shift toward moderate or mild anxiety (**Table 1A**).

### 3.2 Acute anxiolytic effect of exercise

**Figure 1:**
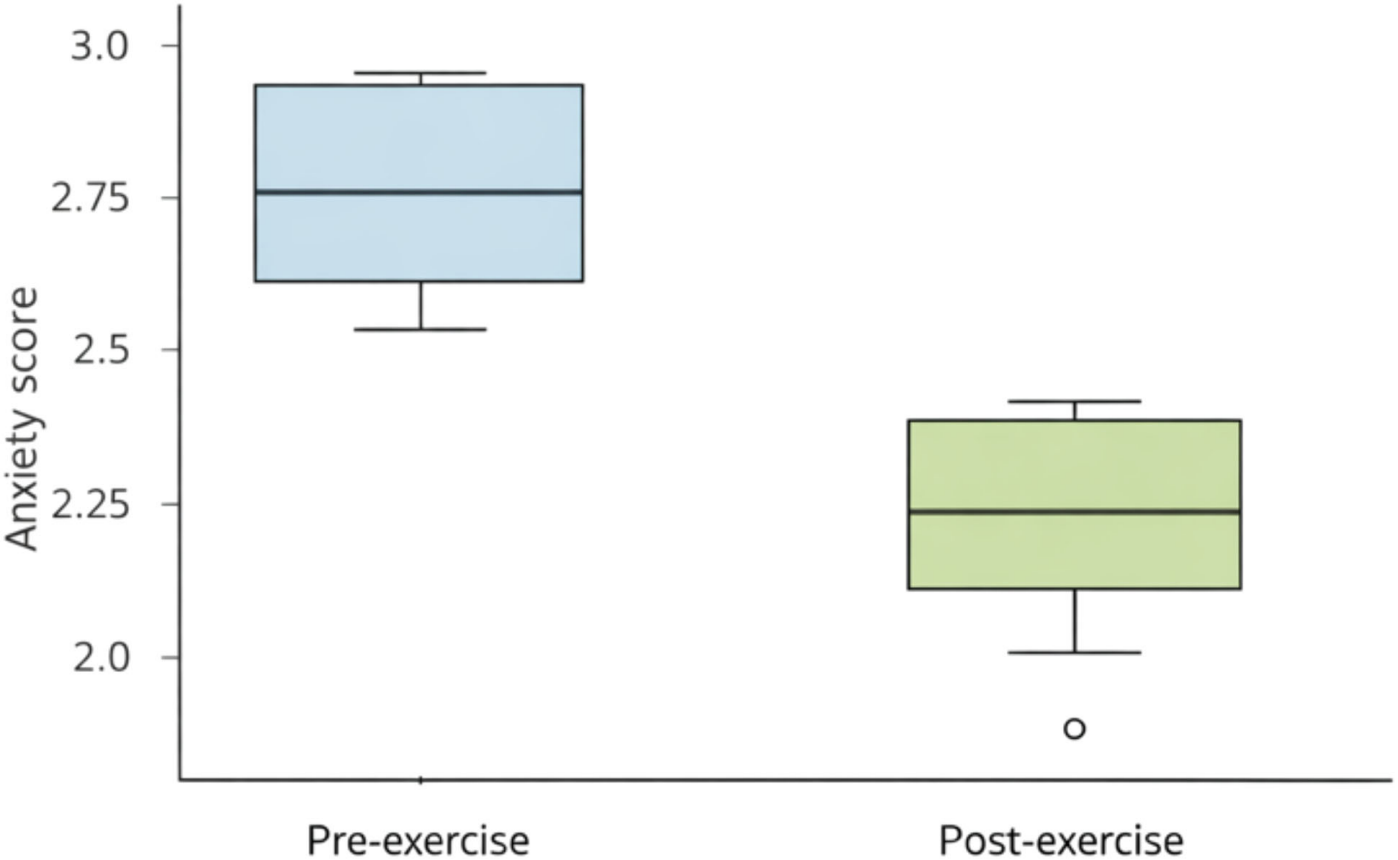
Pre- vs post-exercise self-reported anxiety. Boxplots (median, IQR, and 1.5×IQR whiskers) with overlaid jittered points showing individual pre- and post-exercise self-reported anxiety scores. The distribution shifts downward from severe to moderate/mild levels after a single therapeutic exercise session.

Across all patients, self-reported anxiety decreased reliably from pre- to post-exercise (**Table 2**, **Figure 1**). The median within-person change was −1 category (post − pre), with: (i) 145 patients improved; (ii) 34 showed no change; and (iii) no cases of worsening were observed within the constraints of the categorical scale.

**Table 2:** Inferential tests on self-reported anxiety change and moderators.

Test	Statistic	*P*-value
Wilcoxon signed-rank (pre > post)	W = 10,585	< 0.001
Sign test (improved vs. not improved)	k = 145, n = 179	< 0.001
Mann–Whitney (change by sex)	U = 3630	0.305
Kruskal–Wallis (change by diagnosis)	H = 0.63	0.959
Spearman (age vs. change)	ρ = −0.114	0.129

A paired Wilcoxon signed-rank test (testing whether pre-exercise scores exceeded post-exercise scores) was highly significant (W = 10 585, *P* < 0.001; **Table 2**). A direction-only sign test (testing whether the proportion of improvements > 0.5) also supported a strong anxiolytic effect (k = 145 of 179 improved; *P <* 0.001; **Table 2**). Visual inspection of the pre- and post-exercise distributions showed a systematic downward shift in anxiety scores (**Figure 1**). The within-person reduction was large in magnitude (Wilcoxon Z = 10.45, r = 0.87), indicating a strong shift in self-reported anxiety from pre- to post-session.

### 3.3 Lack of moderation by sex, diagnosis, or age

To examine potential moderators, we analyzed Δ = anxiety_post − anxiety_pre (negative values = improvement). Sex: Women (*n* = 101) and men (*n* = 78) did not differ in the magnitude of self-reported anxiety reduction (Mann–Whitney U = 3630, *P* = 0.305; **Table 2**). Diagnosis: Change scores did not differ across the five diagnostic categories (Kruskal–Wallis H = 0.63, *P* = 0.959; **Table 2**). Age: There was a small, non-significant trend toward greater improvement at higher age (Spearman ρ = −0.114, *P* = 0.129; **Table 2**).

In every sex and diagnosis subgroup, Wilcoxon tests comparing pre- and post-exercise scores indicated significant improvement. Thus, the anxiolytic effect of exercise was transdiagnostic and robust across demographics, with no evidence that any subgroup failed to benefit.

### 3.4 Dose–response association between exercise exposure and anxiety reduction

**Figure 2:**
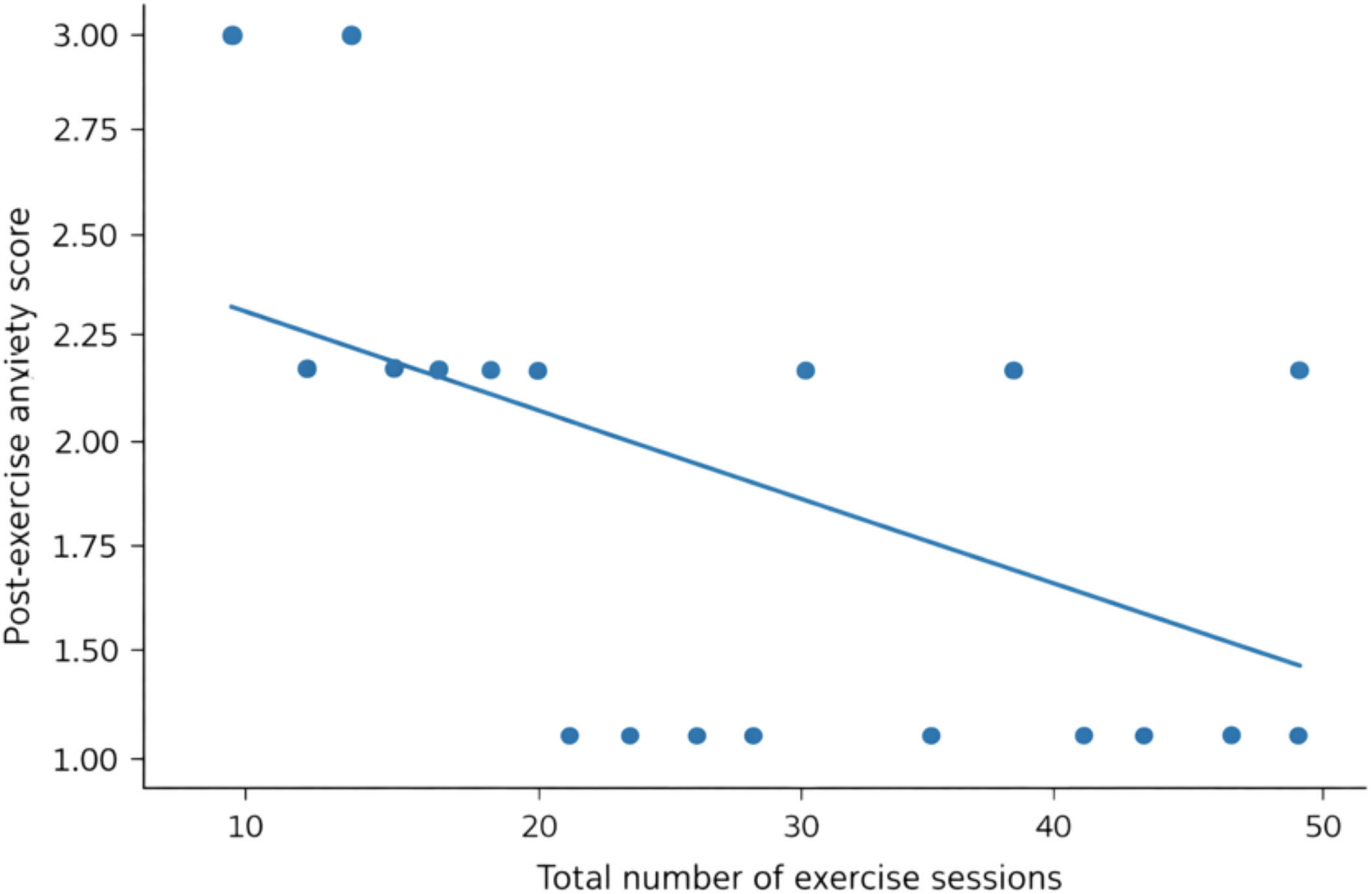
Dose–response between total exercise sessions and post-exercise self-reported anxiety. Scatterplot of post-exercise anxiety as a function of total exercise sessions (sum of walking, stretching/yoga, and breathing sessions), with an overlaid linear regression line. Higher exercise dose is associated with lower post-exercise self-reported anxiety.

Patients varied in the number of sessions completed in each modality: walking (mean 7.8 ± 3.0), stretching/yoga-like exercises (7.9 ± 3.0), and diaphragmatic breathing (7.9 ± 3.0). Spearman correlations revealed strong dose–response associations between session counts and Δ = anxiety_post − anxiety_pre (negative values indicate improvement): walking ρ = −0.628, stretching/yoga ρ = −0.658, and breathing ρ = −0.653 (**Table 3**, **Figure 2**, all P < 0.001).

**Table 3:** Spearman correlations between exercise dose and self-reported anxiety.

Exercise modality	ρ(change, sessions)	*P*(change)	ρ(post, sessions)	*P*(post)	ρ(pre, sessions)	*P*(pre)
Walking sessions	−0.628	< 0.001	−0.557	< 0.001	0.051	0.499
Stretching/yoga sessions	−0.658	< 0.001	−0.582	< 0.001	0.057	0.452
Breathing exercises	−0.653	< 0.001	−0.579	< 0.001	0.054	0.470

Δ = anxiety_post − anxiety_pre (negative values indicate improvement).

Because modality-specific session counts were highly intercorrelated (ρ = 0.97–0.99), we conducted regression analyses using a single composite exposure measure (total sessions = walking + stretching/yoga + breathing) to quantify the dose–response association and to provide effect size estimates with confidence intervals. In an unadjusted linear model, higher total participation was associated with greater improvement (Δ): β = −0.046 per session (95% CI −0.056 to −0.037), R² = 0.429.

In a sensitivity analysis adjusting for age, sex, and diagnosis (robust standard errors), the association was essentially unchanged: β = −0.047 per session (95% CI −0.057 to −0.037). This corresponds to an average additional improvement of −0.47 categories per 10 sessions (95% CI −0.57 to −0.37). The standardized effect size remained large (standardized β ≈ −0.66; partial R² ≈ 0.35).

Consistent results were observed for post-exercise anxiety: in the adjusted model, higher total participation was associated with lower post-exercise anxiety (β = −0.044 per session; 95% CI −0.054 to −0.033; standardized β ≈ −0.57; partial R² ≈ 0.28). Overall, these analyses indicate a strong and quantitatively substantial association between greater overall program engagement and larger reductions in self-reported anxiety.

## 4 Discussion

In this study, we examined short-term changes in self-reported anxiety following structured exercise in a heterogeneous inpatient psychiatric sample. Three principal findings emerged. First, anxiety scores decreased consistently from pre- to post-session. Second, this pattern was observed across diagnostic categories and demographic groups. Third, greater overall participation in the exercise program was associated with larger reductions in self-reported anxiety.

The pre–post shift in anxiety scores was robust and consistent across subgroups. Although immediate anxiolytic effects of physical activity have been documented in general and clinical populations (4, 5, 9, 11, 20), the present findings extend this literature to a diagnostically heterogeneous inpatient psychiatric sample. The magnitude and consistency of the within-person change suggest that structured movement-based sessions are associated with rapid shifts in subjective anxiety states in real-world clinical settings.

However, several measurement considerations are important. Pre-exercise anxiety ratings were highly concentrated at the upper category of the applied severity scale (median 3 [3–3]), indicating restricted baseline variance. This ceiling clustering limits upward variability and may reduce sensitivity for detecting worsening. Although no cases of deterioration were observed, this pattern should be interpreted cautiously, as scale compression may partially account for the absence of worsening. Notably, post-exercise scores demonstrated greater dispersion, indicating retained downward variability.

The transdiagnostic consistency of the observed associations aligns with dimensional frameworks such as the Research Domain Criteria (RDoC), which emphasize shared arousal and regulatory processes across psychiatric conditions (13). Exercise, stretching, and controlled breathing plausibly influence autonomic balance, somatic tension, and interoceptive awareness (11, 16, 21–23). These findings are consistent with emerging integrative models suggesting that exercise may influence neural connectivity, stress-hormone regulation, and emotional resilience across psychiatric populations (24). Nevertheless, because biological markers were not measured, such mechanisms remain theoretical in the present study.

The observed dose–response pattern indicates that greater overall participation in the therapeutic exercise program was associated with larger anxiety reductions. Importantly, exercise modalities were highly intercorrelated, indicating that session counts primarily reflected overall program adherence rather than independent modality-specific exposure. Furthermore, because exercise participation was not randomized, higher engagement may reflect self-selection factors such as motivation, illness severity, therapeutic alliance, or general treatment adherence. Although pre-exercise anxiety was not correlated with session count, unmeasured confounding cannot be excluded. Therefore, the dose–response association should be interpreted as correlational rather than causal.

From a clinical perspective, structured exercise appears feasible and acceptable within inpatient psychiatric care. Group-based walking, stretching, and breathing sessions require minimal equipment and may be integrated into routine schedules. The observed short-term anxiety shifts suggest potential value in using structured movement as an adjunctive strategy during periods of acute distress. However, controlled trials are required to determine the extent to which these associations reflect exercise-specific physiological effects versus contextual influences.

Several limitations warrant consideration. First, the absence of a non-exercise control condition limits causal inference. Without comparison to quiet rest, structured social interaction, or attention-control activities, it is not possible to isolate exercise-specific effects from time effects, regression toward the mean, social interaction, expectancy responses, or Hawthorne effects.

Second, anxiety was assessed using the GAD-7 in a session-based format. Although repeated administration allowed detection of short-term shifts in symptom endorsement, the instrument was originally validated for two-week recall and not for acute state measurement. Future studies should incorporate validated state anxiety scales and physiological markers to more precisely assess acute anxiolytic effects.

Third, baseline anxiety ratings were highly clustered at the upper severity category, suggesting ceiling compression that may limit sensitivity to bidirectional change. Instruments with greater score granularity may improve measurement precision.

Fourth, exercise intensity was monitored clinically using perceived exertion rather than objective physiological metrics (e.g., heart rate, %HRmax), limiting characterization of physiological load.

Fifth, sessions were conducted in small groups, raising the possibility of clustering or shared environmental influences that were not statistically modeled.

Finally, although medication regimens were stable, pharmacological heterogeneity was not analyzed and may have influenced anxiety levels or responsivity to exercise.

Randomized controlled trials incorporating appropriate comparison conditions and experimentally manipulated exercise doses are needed to clarify causality. Future research should also examine mechanistic pathways using autonomic, endocrine, or neurophysiological markers and determine whether repeated acute improvements translate into sustained clinical benefits.

## 5 Conclusion

Therapeutic exercise participation was consistently associated with short-term reductions in self-reported anxiety across multiple psychiatric diagnoses. Greater overall engagement in the structured exercise program was linked to larger reductions in anxiety. Although causal inference is limited by the observational design and absence of a control condition, the findings support the potential value of integrating structured movement-based activities into routine psychiatric care. Randomized controlled trials are required to determine the extent to which these associations reflect exercise-specific physiological effects versus contextual or expectancy influences.

## Data Availability

The datasets presented in this study can be found in online repositories. The names of the repository/repositories and accession number(s) can be found below: 10.5281/zenodo.18499049.
